# Changing landscape of first-line treatment for locally advanced or metastatic urothelial carcinoma: the progression from platinum-based chemotherapy to platinum-free therapy

**DOI:** 10.3389/fimmu.2025.1604395

**Published:** 2025-06-25

**Authors:** Zengguang Liu, Chen Chen, Jiaxin Yin, Xiaofeng Cong, Ziling Liu

**Affiliations:** Cancer Center, The First Hospital of Jilin University, Changchun, China

**Keywords:** locally advanced, metastatic, urothelial carcinoma (UC), first-line therapy, platinum-free, landscape

## Abstract

Urothelial carcinoma (UC) represents the most common pathological type of bladder cancer. For patients with locally advanced or metastatic UC (la/m UC), the standard of care with platinum-based chemotherapy as the cornerstone has greatly improved the survival time. Although the platinum-containing regimens have been established as the first-line therapeutic approach for la/m UC and demonstrate high initial response rates, most patients experience disease recurrence or metastasis shortly after treatment cessation, compounded by the inherent toxicity associated with platinum agents, which collectively pose substantial challenges to long-term patient survival. Moreover, some patients are ineligible to receive a platinum containing therapy, which greatly affects their potential possible benefit. With the success of the EV-302 study, the combination of enfortumab-vedotin (EV) and pembrolizumab has supplanted chemotherapy as the current standard of care for the first-line treatment of la/m UC, thus initiating a treatment paradigm of platinum-free for the first-line treatment of la/m UC. In recent years, the treatment landscape of la/m UC has witnessed remarkable shifts, evolving from traditional chemotherapy to the emerging “chemotherapy-free (platinum-free)” strategy. This article provides a comprehensive review of the historical development and outlook of first-line treatment strategies for la/m UC, including the role of chemotherapy, the rise of platinum-free and its clinical applications. Through this in - depth exploration, the article endeavors to offer readers a holistic understanding of the present treatment panorama for la/m UC, furnishing them with profound insights into the transformative trajectory and emerging trends within la/m UC treatment strategies.

## 1 Introduction

Urothelial cancer (UC) ranks the ninth most common diagnosed cancer worldwide, with 613,791 new cases and 220,349 deaths occurring in 2022 (1). Despite the current pivotal advances in diagnosis and treatment, approximately 11% of patients with UC are diagnosed at an advanced stage and are not amenable to surgical treatment (2). For decades, platinum-based chemotherapeutic agents have been the first-line standard of care for la/m UC, but the median survival is only 14–15 months (3), making it difficult to meet the survival quest of patients.

The use of immune checkpoint inhibitors (ICIs) targeting programmed death 1 or its ligand (PD1/PDL1) in patients with la/m UC is mainly in second-line treatment (4), maintenance therapy after chemotherapy (5) and first-line application in platinum-intolerant patients (6). With the success of the checkmate-901 study, treatment with nivolumab in combination with gemcitabine and cisplatin has become one of the first-line treatment options for la/m UC, extending the median survival to 21.7 months (7). However, considering patients who cannot tolerate cisplatin chemotherapy, this combination therapy greatly limits the possibility of its widespread utilization.

In recent years, antibody-drug conjugates (ADC) drugs have made significant progress in the treatment of la/m UC, and have become one of the emerging options for treatment of la/mUC (8). Enfortumab-vedotin (EV) is one of the most widely used ADC drugs in advanced UC, which has been proven its efficacy in association with pembrolizumab as a first-line therapy (9), beating a platinum-based chemotherapy paradigm that has dominated for nearly 40 years. More importantly, the success of this study makes it possible to achieve chem-free or platinum-free for first-line treatment of the patients with la/m UC, whether platinum-tolerant or platinum-intolerant.

**Figure 1:**
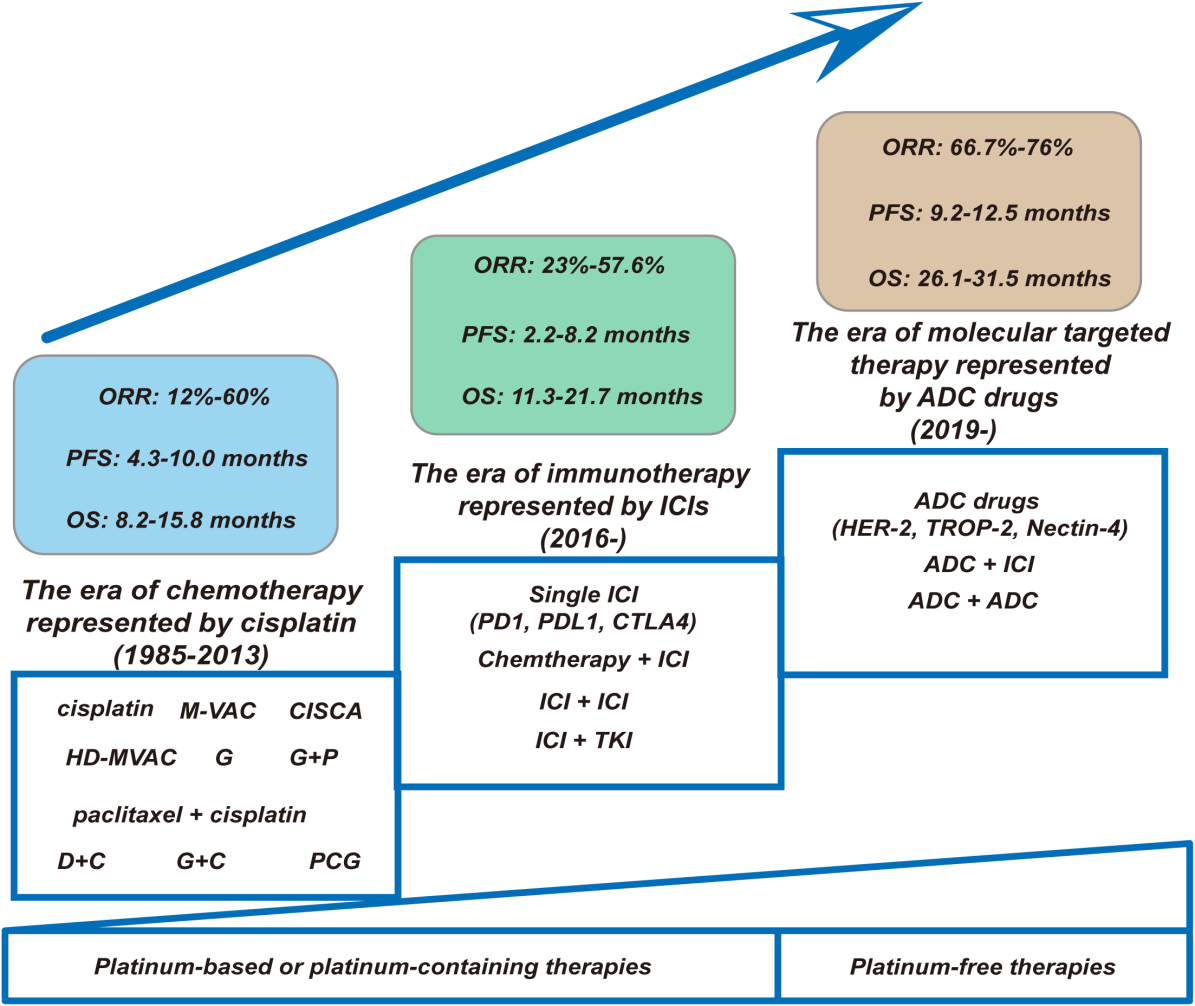
Changing landscape of first-line treatment for la/m UC: the progression from platinum-based chemotherapies or platinum-containing therapies to platinum-free therapies. (1) Chemotherapy Era (1985–2013). This foundational phase was characterized by platinum-based regimens. Key regimens included cisplatin-intensive protocols (M-VAC, CISCA, HD-MVAC), paclitaxel + cisplatin, and non-platinum alternatives (PCG). Despite regimen optimization, therapeutic efficacy remained constrained: ORR ranged modestly from 12–60%, with median PFS of 4.3–10.0 months and OS plateauing at 8.2–15.8 months. (2) Immunotherapy Revolution (2016–). The introduction of ICIs targeting PD-1/PD-L1/CTLA-4 catalyzed a paradigm shift. Monotherapy ICIs were rapidly supplemented by synergistic combinations: chemoimmunotherapy (Chemotherapy + ICI), dual immune blockade (ICI + ICI), and immune-targeted hybrids (ICI + TKI). While ORR improved to 23–57.6%, PFS (2.2–8.2 months) and OS (11.3–21.7 months) exhibited high interpatient variability—reflecting the emergent biomarker-driven selectivity of this approach. (3) Molecular Targeting Era (2019–). ADCs targeting HER-2, TROP-2, and Nectin-4 epitomize contemporary precision oncology. Single-agent ADCs demonstrated remarkable therapeutic promise while combinatorial strategies (ADC + ICI) extended median PFS to 9.2–12.5 months and OS to 26.1–31.5 months. This represents a quantum leap—surpassing chemo-era survival metrics and demonstrating the transformative potential of rational drug engineering. ORR, objective response rate; PFS, progression free time; OS, overall survival; M-VAC, methotrexate, vinblastine, doxorubicin, and cisplatin; CISCA, cisplatin, cyclophosphamide, and adriamycin; HD-MVAC, high-dose-intensity chemotherapy with methotrexate, vinblastine, doxorubicin, and cisplatin plus granulocyte colony-stimulating factor; G, gemcitabine; P, cisplatin; DC, docetaxel and cisplatin; PCG, paclitaxel/cisplatin/gemcitabine; GC, gemcitabine plus cisplatin; ICI, immune checkpoint inhibitor; TKI, tyrosine kinase inhibitor; ADC, antibody-drug conjugate.

Throughout the history of the development of first-line treatment for la/m UC, so far, its development process has mainly experienced the era of chemotherapy represented by cisplatin, the era of immunotherapy represented by ICIs and the era of molecular targeted therapy represented by ADC drugs (as shown in **Figure 1**). This article mainly reviews and elaborates the relevant important studies on the first-line treatment of la/m UC, including the status and current application of cytotoxic chemotherapeutic agents, the specific application of immunotherapy, as well as the emergence of ADC drugs and their application in clinical research, to gain an understanding of the current comprehensive pattern of the first-line treatment of la/m UC.

## 2 The era of chemotherapy laid down the standard first-line treatment based on cisplatin

Despite the challenges in achieving complete remission, cytotoxic chemotherapeutic agents have been playing a pivotal role in the treatment of la/m UC. Among various chemotherapeutic agents, cisplatin-based combination therapies have been extensively investigated (**Table 1**). The methotrexate, vinblastin, adriamycin and cisplatin (M-VAC) regimen was the first polychemotherapy to demonstrate significant efficacy, achieving an overall response rate of 71% and a remarkable complete response rate of 50% in a cohort of 24 patients with advanced UC (10). Based on these findings, subsequent prospective clinical studies on the comparison of M-VAC with cisplatin monotherapy and CISCA combination (cisplatin, cyclophosphamide, adriamycin) have been progressively conducted. M-VAC versus cisplatin monotherapy showed a higher response rate with the M-VAC regimen (39% vs. 12%, P < 0.0001), and the higher response rate in the combination group translated into a significantly longer progression-free survival (PFS) (10.0 vs. 4.3 months) and overall survival (OS) (12.5 vs. 8.2 months) (11). Meanwhile, the results of another prospective clinical study showed that the M-VAC regimen had a better disease remission rate (65% vs. 46%, P < 0.05) and provided longer OS (11.2 vs. 8.4 months) relative to the CISCA regimen (12). Despite the elevated toxicity associated with combination therapy, the positive outcomes of these studies firmly established the M-VAC regimen as the gold standard for first-line treatment of patients with advanced UC (13, 14), based on the high efficacy of the combination and the prolongation of survival. However, long-term follow-up of patients treated with M-VAC revealed that only 3.7% of patients survived and remained disease-free at 6 years. Besides, patients with a poor performance score, and/or bone or visceral metastases were unlikely to benefit significantly from the M-VAC regimen (15).

**Table 1:** Summary of the representative clinical trials of the platinum-containing therapies as the first-line treatment of la/m UC in the era of chemotherapy.

Published year	1992 (11)	1990 (12)	2001 (16) 2006 (17)	1997 (20)	1997 (21)	2000 (22)	1999 (24)	2000 (23)	2000 (3) 2005 (25)	2000 (26)	1999 (27)	2004 (28)	2012 (32)
Intervention	M-VAC *vs.* cisplatin	M-VAC *vs.* CISCA	HD-MVAC *vs.* M-VAC	Single G	Single G	G + P	Weekly G and P	G + P	GP *vs.* MVAC	paclitaxel + cisplatin	Docetaxel and cisplatin	M-VAC *vs.* DC	PCG *vs.* GC
Phase	–	–	III	II	II	II	II	II	III	II	II	III	III
Enrolled patients	269	110	263	40	41	46	42	54	405	34	66	220	626
ORR (%)	39% *vs.* 12% *P* < 0.001	65% *vs.* 46% *P* < 0.05	62% *vs.* 50% *P* = 0.06	28%	24.3%	41%	42%	48%	49% *vs.* 46%	38%	52%	54.2% *vs.* 37.4% *P* = 0.017	55.5% *vs.* 43.6% *P* = 0.003
OS (months)	12.5 *vs.* 8.2	11.2 *vs.* 8.4	15.1 *vs.* 14.9	13.5	8.0	14.3	12.5	13.5	14.0 *vs.* 15.2	Unknown	8.0	14.2 *vs.* 9.3 *P* = 0.026	15.8 *vs.* 12.7 *P* = 0.075
PFS (months)	10.0 *vs.* 4.3	Unknown	9.5 *vs.* 8.1	5.0	Unknown	5.5	7.2	5.75	7.7 *vs.* 8.3	Unknown	5.0	9.4 *vs.* .6.1 *P* = 0.003	8.3 *vs.* 7.6 *P* = 0.11
Grade 3–4 AEs	Unknown	Unknown	Unknown	25%	Unknown	Unknown	78%	40%	71% *vs.* 82%	Unknown	Unknown	35.4% *vs.* 19.2%	35.8% *vs.* 20%

M-VAC, methotrexate, vinblastine, doxorubicin, and cisplatin; CISCA, cisplatin, cyclophosphamide, and adriamycin; HD-MVAC, high-dose-intensity chemotherapy with methotrexate, vinblastine, doxorubicin, and cisplatin plus granulocyte colony-stimulating factor; G, gemcitabine; P, cisplatin; DC, docetaxel and cisplatin; PCG, paclitaxel/cisplatin/gemcitabine; GC, gemcitabine plus cisplatin.

With the advent and utilization of hematopoietic growth factors, studies on modifications to the M-VAC regimen (e.g., administering higher dose of the chemotherapeutic agents, shortening treatment intervals, or substituting other agents in the same class of drugs) have been conducted with the aim of improving the response to treatment and prolonging the survival of patients with la/m UC. A randomized phase III trial comparing high-dose M-VAC (HD-MVAC) with classic M-VAC was conducted by the European Organization for Research and Treatment of Cancer (EORTC), in which the drugs were given on a schedule of every 2 weeks. The results showed that HD-MVAC had a higher overall response (72% vs. 58%) and complete response (25% vs. 11%). Additionally, HD-MVAC significantly improved the PFS (9.5 vs. 8.1 months, P = 0.03) and 2 years PFS rate (24.7% vs. 11.6%) (16). It is worth noting that the systemic use of hematopoietic growth factors made the HD-MVAC be better tolerated. However, after 7.3 years median follow-up, the median OS of different groups was identical (15.1 vs. 14.9 months) (17).

Meanwhile, a few new chemotherapeutic agents including gemcitabine, the taxanes, carboplatin, ifosfamide and vinflunine have been demonstrated activity in UC (18). A series of relevant studies on gemcitabine in UC have yielded good results (19). Two studies investigated the efficacy of gemcitabine alone in the first-line treatment of la/m UC, showing overall response rates of 24.3% and 28%, respectively (20, 21). Encouraged by the potent antitumor capacity and manageable safety profile of gemcitabine alone and previously cisplatin alone, several studies of gemcitabine in combination with cisplatin have been progressively conducted. In a phase II trial of gemcitabine plus cisplatin (GC) in patients with advanced UC (22), results showed that the overall response rate was 41% and the median PFS was 5.5 months and the median OS was 14.3 months. In addition, GC regimen has an acceptable clinical safety profile. Similar results have also been observed in some other Phase II studies (23, 24). These encouraging results have promoted the launch of a Phase III clinical trial of GC regimen versus the M-VAC regimen for the first-line treatment of la/m UC. The trial showed gemcitabine plus cisplatin had a similar OS compared to M-VAC (13.8 vs. 14.8 months, hazards ratio [HR], 1.04; 95% confidence interval [CI], 0.82 to 1.32; P = 0.75), but with better safety profile. Compared with patients received GC therapy, a higher proportion of MVAC patients experienced grade 3/4 neutropenia (82% vs. 71%), neutropenic fever (14% vs. 2%), neutropenic sepsis (12% vs. 1%), grade 3/4 mucositis (22% vs. 1%), and alopecia (55% vs. 11%). During treatment, quality of life was maintained in both groups; however, more patients receiving GC reported better outcomes in terms of weight, performance, and fatigue. Based on the favorable and comprehensive balance of risks and benefits, GC regimen was considered not inferior to MVAC and could be used as a standard alternative therapy to M-VAC (3, 25).

In addition, the use of combination regimens of cisplatin and paclitaxel analogues in the first-line treatment of la/m UC has also been explored. Although cisplatin in combination with paclitaxel or docetaxel regimens achieved objective remission rates of 52-70% in phase II clinical studies (26, 27), the results of a randomized phase III clinical trial comparing docetaxel plus cisplatin (DC) with M-VAC demonstrated that M-VAC was more effective than DC in advanced UC (response rate was 54.2% vs. 37.4%, P = 0.017; median time to progression was 9.4 vs. 6.1 months, P = 0.003; and median survival was 14.2 vs. 9.3 months, P = 0.026) and that the granulocyte colony-stimulating factor (G-CSF) supported M-VAC regimen was better tolerated as an alternative to the classical M-VAC regimen as first-line treatment for la/m UC (28). In this trial, MVAC was more commonly associated with grade 3 or 4 neutropenia (35.4% vs. 19.2%; P = 0.006), thrombocytopenia (5.7% vs. 0.9%; P = 0.046), and neutropenic sepsis (11.6% vs. 3.8%; P = 0.001). The toxicity of MVAC was significantly lower than previously reported for MVAC administered without G-CSF.

During clinical treatment, the tolerance of cisplatin-based drugs in patients with la/m UC is a key consideration in our choice of treatment options. For patients who was ineligible for cisplatin-based chemotherapy, it was fit to choose the carboplatin-based protocols (29). The combination of gemcitabine and carboplatin could avoid the nephrotoxicity often associated with cisplatin. A phase II/III trial conducted by the ETORC comparing the gemcitabine plus carboplatin with carboplatin, methotrexate and vinblastine (M-CAVI) in patients unfit for platinum-based therapy showed that the two groups had an identical OS, response rate and PFS and the combination of gemcitabine and carboplatin had a better toxicity profile (30). But for patients who are fit for cisplatin-based chemotherapy, gemcitabine plus carboplatin cannot replace the gemcitabine and cisplatin regimen (31).

To further improve treatment efficacy, a phase III study was conducted by the EORTC group to test the addition of a third drug to the standard GC regimen. The results showed that the addition of paclitaxel to GC provided a higher response rate (55.5% vs. 43.6%) and a 3.1-month survival benefit (15.8 vs. 12.7 months, HR = 0.85; P = 0.075) that did not reach statistical significance (32). Both treatments were well tolerated. The incidence of thrombocytopenia and bleeding was higher in the GC group than in the PCG group (11.4% vs. 6.8%; P = 0.05), while the incidence of febrile neutropenia was 13.2% vs. 4.3% in the PCG group and GC group, respectively; P < 0.001.

In summary, the results of numerous clinical studies have shown that in the era of chemotherapy, various cisplatin-based chemotherapy regimens (including GC, M-VAC, and HD-MVAC) have laid the cornerstone of first-line treatment for la/m UC. For patients unsuitable for cisplatin, gemcitabine combined with carboplatin or MCAVI regimen is the preferred treatment option. Besides, given the more hospital admissions, the greater medical resources administration and the associated toxicity, the combination of gemcitabine with cisplatin or carboplatin is the preferred choice in practice.

## 3 The era of immunotherapy: cooperative warfare is preferable to fighting alone

The introduction of ICIs, represented by the anti-PD1/PDL1, has brought new options for the treatment of advanced malignancies. For patients with la/m UC, the success of relevant clinical studies of ICIs has also revolutionized the treatment landscape. Here, we mainly review the application of immunotherapy in the first-line treatment of la/m UC (**Table 2**).

**Table 2:** Summary of the representative clinical trials of the immunotherapy -containing therapies as the first-line treatment of la/m UC in the era of immunotherapy.

Published year	2017 (33)	2017 (34) 2020 (35)	2020 (36)	2021 (38)	2023 (7)	2020 (40)	2024 (41)
Study	NCT02108652	KEYNOTE-052 NCT02335424	IMvigor130 NCT02807636	KEYNOTE-361 NCT02853305	CheckMate901 NCT03036098.	DANUBE NCT02516241	LEAP-011
Intervention	T	K	A: T + plt/gem B: T C: plt/gem	A: K + plt/gem B: K C: plt/gem	O + GP *vs.* GP	A: I B: I + tremelimumab → I C: plt/gem	lenvatinib + K *vs.* placebo + K
Phase	II	II	III	III	III	III	III
Enrolled patients	123	374	1213	1010	608	1032	487
ORR (%)	23%	28.6%	47% *vs.* 23% *vs.* 44%	54.7% *vs.* 30.3% *vs.* 44.9%	57.6% *vs.* 43.1%	26% *vs.* 36% *vs.* 49%	33% *vs.* 29%
OS (months)	15.9	11.3	A: C 16.0 *vs.* 13.4 *P* = 0.027 B: C 15.7 *vs.*13.1	A: C 17.0 *vs.* 14.3 *P* = 0.0407 B: C 15.6 *vs.*14.3	21.7 *vs.* 18.9 *P* = 0.02	B: C 15.1 *vs.* 12.1 *P* = 0.075	11.8 *vs.* 12.9
PFS (months)	2.7	2.2	A: C 8.2 *vs.* 6.3 *P* = 0.007	A: C 8.3 *vs.* 7.1 *P* = 0.0003	7.9 *vs.* 7.6 *P* = 0.001	2.3 *vs.* 3.7 *vs.* 6.7	4.5 *vs.* 4.0
Grade 3–4 AEs	16%	20.8%	85% *vs.* 42% *vs.* 86%	96% *vs.* 18% *vs.* 68%	61.8% *vs.* 51.7%	14% *vs.* 27% *vs.* 60%	51% *vs.* 27%
Drug Discontinuation rate due to drug- related AEs	8%	9.2%	34% *vs.* 6% *vs.* 34%	38% *vs.* 10% *vs.* 18%	21.1% *vs.* 17.4%	6% *vs.* 16% *vs.* 12%	20% *vs.* 9%
iRAE	12%	25.9%	50% *vs.* 37%	10% *vs.* 10%	29.3%	18% *vs.* 37%	Unknown

T, atezolizumab; K, pembrolizumab; plt/gem, platinum/gemcitabine; O, nivolumab; GP, gemcitabine and cisplatin; I, durvalumab.

In 2017, atezolizumab and pembrolizumab were the first two ICIs approved by Food & Drug Administration (FDA) as the first-line therapy for the patients with la/m UC unfit for cisplatin-based therapy according to the results of the phase II trials Imvigor-210 and KEYNOTE-052. The IMvigor-210 study (Cohort 1) evaluated the efficacy and safety of atezolizumab as first-line treatment in patients with la/m UC who were not candidates for cisplatin therapy. The results showed that 119 patients enrolled for treatment had an objective response rate (ORR) of 23% at a median follow-up of 17.2 months, including a complete response (CR) rate of 9%, with a median PFS and OS of 2.7 months versus 15.9 months, respectively (33). Among treatment-related adverse events (TRAEs), those with an incidence rate of 10% or higher included fatigue (36 cases, 30%), diarrhea (14 cases, 12%), and pruritus (13 cases, 11%). One treatment-related death (sepsis) occurred. Nine patients (8%) discontinued treatment due to adverse events (AEs). Immune-mediated events occurred in 14 patients (12%). The KEYNOTE-052 study is a single-arm phase II study applying pembrolizumab for the first-line treatment of platinum-intolerant patients. The results of the trial showed an ORR of 24% and a CR rate of 5% among the 370 patients treated (34). Updated data with a median follow-up of 56.3 months were reported at the 2021 ASCO meeting, showing ORR and survival outcomes comparable to previous reports: an ORR of 28.9%, a median duration of efficacy (DOR) of 33.4 months, and a median OS of 11.3 months. Among them, patients with PD-L1-expressing CPS ≥10 had better efficacy, with ORR of 47.3% versus 20.7%, DOR of NR versus 21.2 months, and OS of 18.5 versus 9.7 months, respectively, compared with patients with CPS <10 (35). In terms of safety, 67.3% of patients experienced TRAEs; the most common were fatigue (18.1%) and pruritus (17.8%). 20.8% of patients experienced at least grade 3 TRAEs, with the most common being fatigue (2.4%), colitis (1.9%), elevated serum alkaline phosphatase levels (1.6%), muscle weakness (1.4%), and hepatitis (1.4%). The incidence of immune-mediated AEs (regardless of causality) was 25.9%.

Encouraged by these clinical results, corresponding phase III clinical trials of atezolizumab and pembrolizumab for the first-line treatment of la/m UC were being conducted concurrently (namely Imvigor-130 and KEYNOTE-361). IMvigor-130 is a global, randomized phase III study designed to assess the efficacy of first-line atezolizumab in combination with platinum/gemcitabine (group A), atezolizumab monotherapy (group B), and placebo in combination with platinum/gemcitabine (group C) in patients with metastatic UC. At a median follow-up of 11.8 months, the results of the study showed that the median PFS of group A and group C were 8.2 months and 6.3 months, respectively (one-sided P = 0.007), and the median OS of the two groups were 13.4 months versus 16.0 months, which had not yet reached a statistically set significant difference. In addition, atezolizumab monotherapy (group B) did not improve OS compared with group C, as suggested by the interim analysis (15.7 vs. 13.1 months) (36). At the 2023 ASCO-GU Annual Meeting, IMvigor-130 presented the results of its final OS analysis. OS improvement in the intention-to-treat (ITT) population did not show a statistically significant difference for atezolizumab in combination with platinum/gemcitabine compared to placebo. However, when atezolizumab monotherapy was compared with chemotherapy for first-line treatment of la/m UC, atezolizumab monotherapy demonstrated better tolerability than chemotherapy (37). The most common Grade 3–4 TRAEs were anemia (168 of 454 patients [37%] receiving atezolizumab plus chemotherapy versus 133 of 389 patients [34%] receiving placebo plus chemotherapy), neutropenia (167 [37%] versus 115 [30%]), thrombocytopenia (95 cases [21%] versus 70 cases [18%]), and decreased platelet count (92 cases [20%] versus 92 cases [24%]). Serious adverse events occurred in 243 patients (54%) receiving atezolizumab plus chemotherapy and 196 patients (50%) receiving placebo plus chemotherapy. Treatment-related deaths occurred in 9 patients receiving atezolizumab plus chemotherapy and 4 patients who received placebo plus chemotherapy.

The phase III KEYNOTE-361 study enrolled 1010 patients with untreated advanced, unresectable or metastatic uroepithelial carcinoma who were randomized into 3 groups and given pembrolizumab alone, pembrolizumab in combination with chemotherapy (gemcitabine plus cisplatin or carboplatin), and chemotherapy. The primary endpoints of the study were OS and PFS. The results of the study at a median follow-up time of 31.7 months showed that the addition of pembrolizumab to chemotherapy could not significantly prolong median PFS or OS. Moreover, OS data were similar in both groups with no statistical difference when chemotherapy was compared with immunological monotherapy, regardless of whether the whole population or the population with high PDL1 expression at the time (38). It is also important to note that during the KEYNOTE-361 and IMvigor-130 studies, a safety warning was issued by the FDA regarding the shortened survival time observed with first-line ICIs monotherapy versus first-line chemotherapy. According to the latest NCCN guidelines (39), pembrolizumab is recommended for the first-line treatment of patients with la/m UC who cannot tolerate cisplatin-containing chemotherapy and atezolizumab is considered as a first-line option for patients whose tumors express PD-L1 or who are not eligible for any platinum-containing chemotherapy regardless of PD-L1 expression.

Although the combination of pembrolizumab or atezolizumab with chemotherapy has failed to change the first-line treatment paradigm for la/m UC, this treatment paradigm based on chemotherapy in combination with immunotherapy has not been put on hold in the first-line treatment of la/m UC. The CheckMate-901 study was a phase III, randomized, open-label trial evaluating the addition of nivolumab to gemcitabine-cisplatin (GC) versus GC chemotherapy for the first-line treatment of cisplatin-tolerant la/mUC, with the primary endpoints of OS and PFS. Results showed that at a median follow-up of 33.6 months, the nivolumab combination therapy could significantly prolong the PFS (7.9 vs. 7.6 months, HR for progression or death, 0.72; 95% CI, 0.59 to 0.88; P = 0.001) and OS (21.7 vs. 18.9 months, HR for death, 0.78; 95% [CI], 0.63 to 0.96; P = 0.02) (7). 99.7% of patients in the combination therapy group and 98.6% of patients in the chemotherapy group experienced adverse events of any cause; adverse events of grade 3 or higher occurred in 61.8% and 51.7% of patients, respectively. TRAEs of any grade leading to discontinuation occurred in 21.1% of patients in the combination group and 17.4% of patients in the chemotherapy group; the corresponding percentages for grade 3 or higher AEs leading to discontinuation were 11.2% and 7.6%, respectively. Overall health status as assessed by the EORTC QLQ-C30 remained stable in both groups. Based on the success of the CheckMate-901 study, the regimen of nivolumab, gemcitabine and cisplatin followed by nivolumab maintenance is recommended as first-line therapy for la/m UC.

It is not known why only Checkmate-901 was successful in a similar trial design, and a combined analysis of the enrolment populations of the three clinical studies suggests that one possible reason is that the Checkmate-901 study enrolled patients who were in better general condition and who were more tolerant of cisplatin-based chemotherapy. This is because the only platinum used in the study was cisplatin.

Besides, the combination of different ICIs and the combination of ICIs and tyrosine kinase inhibitors (TKIs) have been explored in the first-line treatment of la/m UC, but the results are not satisfactory, and the ideal of first-line de-chemotherapy for la/m UC cannot be achieved yet. The phase III clinical study DANUBE explored the combination of durvalumab and tremelimumab versus chemotherapy in the first-line treatment of la/m UC, and showed that the ICI/ICI combination treatment did not improve survival compared to chemotherapy (15.1 vs. 12.1 months, P = 0.075) (40). Another Phase III clinical study, LEAP-011, evaluated pembrolizumab in combination with lenvatinib or placebo for the first-line treatment of patients with platinum-intolerant advanced UC and showed that the addition of lenvatinib to pembrolizumab was not superior to pembrolizumab monotherapy, with an identical PFS (4.5 vs. 4.0 months, HR 0.90 [95% CI 0.72-1.14]) and OS (11.8 vs. 12.9 months, HR 1.14 [95% CI 0.87-1.48]) (41).

In summary, single ICI, or the ICI/ICI combination therapy as well as ICI combined with TKIs, have not yet enabled de-chemotherapy for the first-line treatment of la/m UC. For those who cannot tolerate platinum-based therapy, single immunotherapy may be a first-line alternative, and the success of the Checkmate-901 study suggests that gemcitabine plus cisplatin in combination with nivolumab followed by nivolumab maintenance may be the standard first-line treatment for patients with la/m UC in those who can tolerate cisplatin-based chemotherapy.

## 4 The era of ADC medication enables first-line platinum-free for patients with la/m UC

ADC drugs consist of a monoclonal antibody targeting a specific antigen, a highly potent cytotoxic drug, and a chemical linker connecting the two (42), thus enabling highly targeted delivery of loaded drugs to tumors while reducing the side effects of the conventional use of chemotherapeutic drugs (43). However, the challenges posed by drug resistance may result in patients treated with ADC alone eventually experiencing disease progression (44). Therefore, to achieve better outcomes in first-line therapy and to overcome the occurrence of drug resistance, combination therapy based on ADC drugs is currently the main option in clinical studies (45, 46). In clinical studies related to UC, the combination of ADC drugs and immune checkpoint inhibitors is currently the most promising strategy. Nowadays, the more widely clinical studied ADC agents in UC include ADC drugs targeting Nectin-4, human epidermal growth factor receptor 2 (HER-2) and trophoblast cell surface antigen 2 (TROP-2). Here, we mainly review the application of ADC drugs in the frontline therapy of la/m UC (**Table 3**).

**Table 3:** Summary of the representative clinical trials of the ADC medication containing therapies as the first-line treatment of la/m UC in the era of ADC drugs.

Published year	2023 (47)	2024 (9)	2023 (51)
Study	EV103	EV302	RC48-C014
Intervention	EV + K	EV + K *vs.* plt/gem	RC48-ADC + toripalimab
Phase	Ib/II	III	Ib/II
Enrolled patients	45	886	41
ORR (%)	73.3%	66.7% *vs.* 44.4%	76%
OS (months)	26.1	31.5 *vs.* 16.1 *P* < 0.001	Unkonwn
PFS (months)	12.3	12.5 vs. 6.3 P < 0.001	9.2
Grade 3–4 AEs	64.4%	55.9% *vs.* 69.5%	43.9%
Drug Discontinuation rate due to drug- related AEs	24.4%	35% *vs.*18.5%	Unkonwn

The EV-103 study was an open-label, multi-cohort that first explored the efficacy and safety of EV in combination with pembrolizumab in patients with la/mUC who were intolerant to cisplatin and had not been treated with systemic therapy before. Forty-five patients with la/m UC were treated with the EV plus pembrolizumab therapy and the results showed that all subjects had an investigator-assessed ORR of 73.3%, a median PFS of 12.3 months, and a median OS of 26.1 months (47). The most common TRAEs were peripheral sensory neuropathy (55.6%), fatigue (51.1%), and alopecia (48.9%). Twenty-nine patients (64.4%) experienced TRAEs of grade 3 or higher; the most common were elevated lipase (17.8%), macular papules (11.1%), and fatigue (11.1%). One death (2.2%) was classified as a TRAE. Based on the results of the trial, EV plus pembrolizumab was acceleratedly approved by the FDA as the first-line treatment for patients with la/m UC who are cisplatin-ineligible (48).

The phase III EV-302/KEYNOTE-A39 clinical trial enrolled 886 la/m UC patients to further evaluate EV plus pembrolizumab against platinum-based chemotherapy (gemcitabine in combination with cisplatin or carboplatin) regardless of the patients’ PDL-1 expression and cisplatin eligibility (9). Results of the trial indicated that the combination therapy could markedly improve PFS (12.5 vs. 6.3 months; HR = 0.45, 95% [CI]: 0.38-0.54; P < 0.001) and OS (31.5 vs. 16.1 months; HR = 0.47, 95% [CI]: 0.38-0.58; P < 0.001) compared to chemotherapy at a median duration of follow-up for 17.2 months. Besides, the new regimen had a higher confirmed ORR of 66.7% with no new safety signals reported. According to the exciting results of the trial, the FDA granted traditional approval to EV plus pembrolizumab for patients with la/m UC (49). The most common TRAEs in the combined group at any grade were peripheral sensory neuropathy (50.0%), pruritus (39.8%), and alopecia (33.2%); in the chemotherapy group, the most common such events were anemia (56.6%), neutropenia (41.6%), and nausea (38.8%). The incidence rates of TRAEs of grade 3 or higher were 55.9% in the experimental group and 69.5% in the control group. The most common grade 3 or higher AEs in the combination group were maculopapular rash (7.7% of patients), hyperglycemia (5.0%), and neutropenia (4.8%); in the chemotherapy group, they were anemia (31.4%), neutropenia (30.0%), and thrombocytopenia (19.4%). TRAEs leading to discontinuation of any treatment occurred in 35.0% and 18.5% of patients, respectively. The most common TRAEs of the two groups leading to discontinuation of any study drug were peripheral sensory neuropathy (10.7% of patients) and anemia (2.8%). Based on the overall success of the study, both the NCCN guidelines (39) and the ESMO guidelines (50) have adopted the treatment regimen of EV in combination with pembrolizumab as the first-line standard of care for la/m UC.

Another ADC drug that has been studied extensively in UC is the one targeting HER-2, represented by disitamab vedotin (RC48-ADC). RC48-C014 was the first trial to study the RC48-ADC in combination with toripalimab (anti-PD1) in 41 patients enrolled with la/m UC in China. Results indicated that the combination therapy as a first-line utilization had an ORR of 76%, including 10% CR, with a median PFS of 9.2 months and 2-year OS rate of 63.2%. Notably, ORR of 64.3% and 33.3% were also observed in those with low or negative HER-2 expression, respectively (51). The most common TRAEs were elevated AST/ALT (68.3%), peripheral sensory neuropathy (61.0%), weakness (61.0%), elevated γ-glutamyltransferase (56.1%), hypertriglyceridemia (53.7%), and decreased appetite (51.2%). Grade 3 or higher TRAEs occurred in 43.9% of patients. Twenty-three patients (56.1%) experienced grade 3 or higher immune-related adverse events. This study provided the initial evidence of the efficacy and safety of this combination therapy for further dissemination in la/m UC. Subsequently, a randomized phase III trial comparing the combination of RC48-ADC with toripalimab to standard chemotherapy for first-line treatment of patients with la/mUC expressing HER-2 is ongoing. Additionally, to further expand the beneficiary population of the RC-48-based regimen in la/m UC, RC48G001 (phase II) and SGNDV-001 (phase III) are ongoing to evaluate the efficacy of RC48-ADC combined with pembrolizumab.

Sacituzumab govitecan-hziy (SG) was a novel ADC which targets TROP-2. TROPHY-U-01 first investigated the efficacy of SG in patients with la/m UC who failed to platinum-based chemotherapy and ICIs. An updated results showed that ORR was 28%, median PFS and median OS were 5.4 months and 10.9 months, separately. And SG displayed a manageable safety profile which was consistent the reported data (52). A phase III study (NCT06524544) comparing the combination of pembrolizumab and SG versus SG alone in the frontline treatment of la/m UC is ongoing.

## 5 Future perspectives in first-line treatment of la/m UC: precision and combination

With the wide application of genetic testing technology, the future first-line treatment of la/m UC tends to further precise combination of treatment modes. By searching in the Web of Clinical Trials (https://clinicaltrials.gov/), it is found that the first-line treatment of la/m UC in the future is still in the direction of ICIs combined with ADC drugs, where the targets of ADC drugs include Nectin4 (NCT06225596, NCT06592326), HER2 (NCT06178601, NCT04879329, NCT05911295 and NCT05302284), Trop2 (NCT06524544) and EGFR-HER3 (NCT06405425). The success of the EV302 study has created a new platinum-free therapy (chemo-free) treatment model for the first-line treatment of la/m UC. This treatment paradigm has the potential to benefit more patients. In addition, encouraged by the outstanding efficacy of FGFR2/3 inhibitors in the second line of advanced uroepithelial cancer (53), molecularly-targeted drug-based combination therapies for specific targets, mainly FGFR inhibitors in combination with immune-checkpoints, are also being investigated (NCT05775874, NCT03473743). In addition, although the KEYNOTE-361 study and the Imvigor-130 study failed, studies related to this chemotherapy-based model in combination with ICIs are also still ongoing in the first-line treatment of la/m UC (NCT03967977, NCT03682068 and NCT04568304). In addition to two-agent combinations, studies of three-agent combinations based on different therapeutic mechanisms as first-line therapy for la/m/UC have also been conducted (NCT03547973) (**Table 4**)

**Table 4:** Summary of the ongoing first-line therapy clinical trials of la/m UC.

Treatment mode	Clinical trial	Phase	Enrollment patients (N)	Treatment arms	Primary endpoint	Estimated completion date
ADC + IO	Duravelo-2 (NCT06225596)	2/3	956	BT8009 *vs.* BT8009 + K	PFS	2030.12
	NCT06592326	3	460	9MW2821+Toripalimab *vs.* standard chemotherapy	PFS, OS	2028.12
	NCT06405425	2	52	BL-B01D1 + PD-1	ORR	2026.5
	NCT06178601	2	36 (HER-2+)	RC48-ADC + AK104	ORR	2026.4.1
	NCT04879329	2	332 (HER-2+)	Cohort C Disitamab vedotin monotherapy Cohort E Disitamab vedotin + K	ORR	2028.5.30
	NCT05911295	3	700 (HER-2+)	Disitamab vedotin + K *vs.* standard chemotherapy	PFS OS	2029.4.30
	NCT05302284	3	452 (HER-2+)	Disitamab vedotin + Toripalimab *vs.* standard chemotherapy	PFS OS	2028.4.30
	NCT06524544	3	384	K +SG *vs.* to SG	OS	2028.12.31
Molecularly targeted drug-based therapy	NCT05775874	2	80	AZD4547 + Tislelizumab (FGFR2/3 alterations)	ORR	2025.12.30
	NCT03473743	1/2	120	Erdafitinib alone *vs.* erdafitinib + cetrelimab (FGFR alterations)	ORR	2025.6.30
	NCT03375307	2	150	Olaparib (DNA-repair genetic changes)	ORR	2025.12.16
Chemotherapy + IO	NCT03967977	3	420	Tislelizumab + standard chemotherapy *vs.* placebo+ standard chemotherapy	OS	2027.06
	NCT03682068	3	1246	Durvalumab ± tremelimumab with chemotherapy followed by durvalumab monotherapy *vs.* chemotherapy	OS	2025.6.30
	NCT04568304	3	364	Toripalimab + standard chemotherapy *vs.* placebo+ standard chemotherapy	PFS	2025.11.30
Other	NCT03547973	2	827	Cohort 4: SG + Cisplatin + Avelumab Cohort 7: arm 1 SG + EV + ZIM arm 2 EV + ZIM	ORR	2030.6

ADC, antibody–drug conjugate; BT8009, A nectin-4 targeting bicycle toxin conjugate; K, pembrolizumab; 9MW2821, a nectin-4-targeting antibody–drug conjugate; BL-B01D1, a first-in-class EGFR–HER3 bispecific antibody–drug conjugate; RC48-ADC (Disitamab vedotin), a novel HER2-targeting antibody–drug conjugate; AK104, a PD-1/CTLA-4 bispecific antibody; SG, sacituzumab govitecan, a TOP2-targeting antibody–drug conjugate; AZD4547, a multikinase inhibitor of the FGFR1–3 kinases; cetrelimab, an anti–PD-1 monoclonal antibody; Standard chemotherapy, Gemcitabine + Cisplatin/Carboplatin, ZIM, Zimberelimab, an anti–PD-1 monoclonal antibody.

Tailoring treatment plans based on a patient’s specific tumor biology characteristics (such as molecular typing, gene mutations, expression profiles, and immune microenvironment) is a key direction for improving efficacy, reducing toxic side effects, and improving patient quality of life. With the advancement of genetic testing technology and the maturation of more refined stratification for urothelial carcinoma, we believe that future clinical studies targeting the tumor biology and gene expression profiles of patients will more accurately reflect the personalized and refined treatment of urothelial carcinoma.

## 6 Conclusion

In summary, the success of the EV-302/KEYNOTE-A39 study will change the existing treatment paradigm for la/m UC, and the EV plus pembrolizumab regimen establishes a new standard for first-line treatment of la/m UC, making first-line chem-free of the entire population of la/m UC possible. In addition, relevant clinical studies based on RC48-ADC and SG are expected to provide new options for de-chemotherapy for first-line treatment of la/m UC while improving efficacy.

Throughout the nearly 40-year history of the development of first-line treatment for la/m UC, it has experienced the transformation from traditional platinum-based chemotherapy to platinum-free therapy, obtaining an overall improvement in ORR, PFS and OS. Along with the refinement of relevant molecular diagnostics, the treatment of UC will gradually move towards precision in the future. The comprehensive success of EV plus pembrolizumab has revolutionized the treatment options of la/m UC. Although the combination of EV with pembrolizumab has been recommended as the standard first-line treatment for all populations (platinum-resistant and platinum-intolerant) with la/m UC, it does not mean that chemotherapy has been completely abandoned. Considering various factors such as drug accessibility and economic burden of treatment, platinum-based combination therapy may still be one of the most important options for physicians or patient populations. The transition from platinum-based chemotherapy to platinum-free therapy is not an “all-or-nothing” choice, but rather the result of precise stratification based on biomarkers. In the future, first-line therapy of la/m UC should be centered on “tailored treatment”, balancing group data with individual differences, and driving the transition from traditional “evidence-based medicine” to precision-guided “evidence-based medicine”.

## References

[1] Bray F Laversanne M Sung H Ferlay J Siegel RL Soerjomataram I. Global cancer statistics 2022: GLOBOCAN estimates of incidence and mortality worldwide for 36 cancers in 185 countries. CA Cancer J Clin. (2024) 74:229–63. doi: 10.3322/caac.21834 38572751

[2] Geynisman DM Broughton E Hao Y Zhang Y Le T Huo S. Real-world treatment patterns and clinical outcomes among patients with advanced urothelial carcinoma in the United States. Urol Oncol. (2022) 40:195.e1–195. doi: 10.1016/j.urolonc.2021.11.014 34906410

[3] von der Maase H Hansen SW Roberts JT Dogliotti L Oliver T Moore MJ. Gemcitabine and cisplatin versus methotrexate, vinblastine, doxorubicin, and cisplatin in advanced or metastatic bladder cancer: results of a large, randomized, multinational, multicenter, phase III study. J Clin Oncol. (2000) 18:3068–77. doi: 10.1200/JCO.2000.18.17.3068 11001674

[4] Bellmunt J de Wit R Vaughn DJ Fradet Y Lee JL Fong L. Pembrolizumab as second-line therapy for advanced urothelial carcinoma. N Engl J Med. (2017) 376:1015–26. doi: 10.1056/NEJMoa1613683 PMC5635424 28212060

[5] Grivas P Park SH Voog E Caserta C Gurney H Bellmunt J. Avelumab first-line maintenance therapy for advanced urothelial carcinoma: comprehensive clinical subgroup analyses from the JAVELIN bladder 100 phase 3 trial. Eur Urol. (2023) 84:95–108. doi: 10.1016/j.eururo.2023.03.030 37121850

[6] Suzman DL Agrawal S Ning YM Maher VE Fernandes LL Karuri S. FDA approval summary: atezolizumab or pembrolizumab for the treatment of patients with advanced urothelial carcinoma ineligible for cisplatin-containing chemotherapy. Oncologist. (2019) 24:563–9. doi: 10.1634/theoncologist.2018-0084 PMC6459239 30541754

[7] van der Heijden MS Sonpavde G Powles T Necchi A Burotto M Schenker M. Nivolumab plus gemcitabine-cisplatin in advanced urothelial carcinoma. N Engl J Med. (2023) 389:1778–89. doi: 10.1056/NEJMoa2309863 PMC12314471 37870949

[8] Roviello G Santoni M Sonpavde GP Catalano M. The evolving treatment landscape of metastatic urothelial cancer. Nat Rev Urol. (2024) 21:580–92. doi: 10.1038/s41585-024-00872-0 38702396

[9] Powles T Valderrama BP Gupta S Bedke J Kikuchi E Hoffman-Censits J. Enfortumab vedotin and pembrolizumab in untreated advanced urothelial cancer. N Engl J Med. (2024) 390:875–88. doi: 10.1056/NEJMoa2312117 38446675

[10] Sternberg CN Yagoda A Scher HI Watson RC Ahmed T Weiselberg LR. Preliminary results of M-VAC (methotrexate, vinblastine, doxorubicin and cisplatin) for transitional cell carcinoma of the urothelium. J Urol. (1985) 133:403–7. doi: 10.1016/s0022-5347(17)48996-8 4038749

[11] Loehrer PJ Sr Einhorn LH Elson PJ Crawford ED Kuebler P Tannock I. A randomized comparison of cisplatin alone or in combination with methotrexate, vinblastine, and doxorubicin in patients with metastatic urothelial carcinoma: a cooperative group study. J Clin Oncol. (1992) 10:1066–73. doi: 10.1200/JCO.1992.10.7.1066 1607913

[12] Logothetis CJ Dexeus FH Finn L Sella A Amato RJ Ayala AG. A prospective randomized trial comparing MVAC and CISCA chemotherapy for patients with metastatic urothelial tumors. J Clin Oncol. (1990) 8:1050–5. doi: 10.1200/JCO.1990.8.6.1050 2189954

[13] Calabrò F Sternberg CN. New drugs and new approaches for the treatment of metastatic urothelial cancer. World J Urol. (2002) 20:158–66. doi: 10.1007/s00345-002-0275-2 12196899

[14] Lehmann J Retz M Stöckle M. Is there standard chemotherapy for metastatic bladder cancer? Quality of life and medical resources utilization based on largest to date randomized trial. Crit Rev Oncol Hematol. (2003) 47:171–9. doi: 10.1016/s1040-8428(03)00080-5 12900010

[15] Saxman SB Propert KJ Einhorn LH Crawford ED Tannock I Raghavan D. Long-term follow-up of a phase III intergroup study of cisplatin alone or in combination with methotrexate, vinblastine, and doxorubicin in patients with metastatic urothelial carcinoma: a cooperative group study. J Clin Oncol. (1997) 15:2564–9. doi: 10.1200/JCO.1997.15.7.2564 9215826

[16] Sternberg CN de Mulder PH Schornagel JH Théodore C Fossa SD van Oosterom AT. Randomized phase III trial of high-dose-intensity methotrexate, vinblastine, doxorubicin, and cisplatin (MVAC) chemotherapy and recombinant human granulocyte colony-stimulating factor versus classic MVAC in advanced urothelial tract tumors: European Organization for Research and Treatment of Cancer Protocol no. 30924. J Clin Oncol. (2001) 19:2638–46. doi: 10.1200/JCO.2001.19.10.2638 11352955

[17] Sternberg CN de Mulder P Schornagel JH Theodore C Fossa SD van Oosterom AT. Seven year update of an EORTC phase III trial of high-dose intensity M-VAC chemotherapy and G-CSF versus classic M-VAC in advanced urothelial tract tumours. Eur J Cancer. (2006) 42:50–4. doi: 10.1016/j.ejca.2005.08.032 16330205

[18] Perabo FG Müller SC. New agents for treatment of advanced transitional cell carcinoma. Ann Oncol. (2007) 18:835–43. doi: 10.1093/annonc/mdl331 17018703

[19] Bellmunt J Albiol S de Olano AR Pujadas J Maroto P. Spanish Oncology Genitourinary Group (SOGUG). Gemcitabine in the treatment of advanced transitional cell carcinoma of the urothelium. Ann Oncol. (2006) 17 Suppl 5:v113–7. doi: 10.1093/annonc/mdj964 16807437

[20] Stadler WM Kuzel T Roth B Raghavan D Dorr FA. Phase II study of single-agent gemcitabine in previously untreated patients with metastatic urothelial cancer. J Clin Oncol. (1997) 15:3394–8. doi: 10.1200/JCO.1997.15.11.3394 9363871

[21] Moore MJ Tannock IF Ernst DS Huan S Murray N. Gemcitabine: a promising new agent in the treatment of advanced urothelial cancer. J Clin Oncol. (1997) 15:3441–5. doi: 10.1200/JCO.1997.15.12.3441 9396395

[22] Kaufman D Raghavan D Carducci M Levine EG Murphy B Aisner J. Phase II trial of gemcitabine plus cisplatin in patients with metastatic urothelial cancer. J Clin Oncol. (2000) 18:1921–7. doi: 10.1200/JCO.2000.18.9.1921 10784633

[23] Lorusso V Manzione L De Vita F Antimi M Selvaggi FP De Lena M. Gemcitabine plus cisplatin for advanced transitional cell carcinoma of the urinary tract: a phase II multicenter trial. J Urol. (2000) 164:53–6. doi: 10.1016/S0022-5347(05)67447-2 10840423

[24] von der Maase H Andersen L Crinò L Weinknecht S Dogliotti L. Weekly gemcitabine and cisplatin combination therapy in patients with transitional cell carcinoma of the urothelium: a phase II clinical trial. Ann Oncol. (1999) 10:1461–5. doi: 10.1023/a:1008331111654 10643537

[25] von der Maase H Sengelov L Roberts JT Ricci S Dogliotti L Oliver T. Long-term survival results of a randomized trial comparing gemcitabine plus cisplatin, with methotrexate, vinblastine, doxorubicin, plus cisplatin in patients with bladder cancer. J Clin Oncol. (2005) 23:4602–8. doi: 10.1200/JCO.2005.07.757 16034041

[26] Burch PA Richardson RL Cha SS Sargent DJ Pitot HC4 Kaur JS. Phase II study of paclitaxel and cisplatin for advanced urothelial cancer. J Urol. (2000) 164:1538–42. doi: 10.1016/S0022-5347(05)67023-1 11025699

[27] Dimopoulos MA Bakoyannis C Georgoulias V Papadimitriou C Moulopoulos LA Deliveliotis C. Docetaxel and cisplatin combination chemotherapy in advanced carcinoma of the urothelium: a multicenter phase II study of the Hellenic Cooperative Oncology Group. Ann Oncol. (1999) 10:1385–8. doi: 10.1023/a:1008379500436 10631471

[28] Bamias A Aravantinos G Deliveliotis C Bafaloukos D Kalofonos C Xiros N. Docetaxel and cisplatin with granulocyte colony-stimulating factor (G-CSF) versus MVAC with G-CSF in advanced urothelial carcinoma: a multicenter, randomized, phase III study from the Hellenic Cooperative Oncology Group. J Clin Oncol. (2004) 22:220–8. doi: 10.1200/JCO.2004.02.152 14665607

[29] Waxman J Barton C. Carboplatin-based chemotherapy for bladder cancer. Cancer Treat Rev. (1993) 19 Suppl C:21–5. doi: 10.1016/0305-7372(93)90044-r 8221712

[30] De Santis M Bellmunt J Mead G Kerst JM Leahy M Maroto P. Randomized phase II/III trial assessing gemcitabine/carboplatin and methotrexate/carboplatin/vinblastine in patients with advanced urothelial cancer who are unfit for cisplatin-based chemotherapy: EORTC study 30986. J Clin Oncol. (2012) 30:191–9. doi: 10.1200/JCO.2011.37.3571 PMC3255563 22162575

[31] Dogliotti L Cartenì G Siena S Bertetto O Martoni A Bono A. Gemcitabine plus cisplatin versus gemcitabine plus carboplatin as first-line chemotherapy in advanced transitional cell carcinoma of the urothelium: results of a randomized phase 2 trial. Eur Urol. (2007) 52:134–41. doi: 10.1016/j.eururo.2006.12.029 17207911

[32] Bellmunt J von der Maase H Mead GM Skoneczna I De Santis M Daugaard G. Randomized phase III study comparing paclitaxel/cisplatin/gemcitabine and gemcitabine/cisplatin in patients with locally advanced or metastatic urothelial cancer without prior systemic therapy: EORTC Intergroup Study 30987. J Clin Oncol. (2012) 30:1107–13. doi: 10.1200/JCO.2011.38.6979 PMC3341152 22370319

[33] Balar AV Galsky MD Rosenberg JE Powles T Petrylak DP Bellmunt J. Atezolizumab as first-line treatment in cisplatin-ineligible patients with locally advanced and metastatic urothelial carcinoma: a single-arm, multicentre, phase 2 trial. Lancet. (2017) 389:67–76. doi: 10.1016/S0140-6736(16)32455-2 27939400 PMC5568632

[34] Balar AV Castellano D O’Donnell PH Grivas P Vuky J Powles T. First-line pembrolizumab in cisplatin-ineligible patients with locally advanced and unresectable or metastatic urothelial cancer (KEYNOTE-052): a multicentre, single-arm, phase 2 study. Lancet Oncol. (2017) 18:1483–92. doi: 10.1016/S1470-2045(17)30616-2 28967485

[35] Vuky J Balar AV Castellano D O’Donnell PH Grivas P Bellmunt J. Long-term outcomes in KEYNOTE-052: phase II study investigating first-line pembrolizumab in cisplatin-ineligible patients with locally advanced or metastatic urothelial cancer. J Clin Oncol. (2020) 38:2658–66. doi: 10.1200/JCO.19.01213 32552471

[36] Galsky MD Arija JÁA Bamias A Davis ID De Santis M Kikuchi E. Atezolizumab with or without chemotherapy in metastatic urothelial cancer (IMvigor130): a multicentre, randomised, placebo-controlled phase 3 trial. Lancet. (2020) 395:1547–57. doi: 10.1016/S0140-6736(20)30230-0 32416780

[37] Grande E Arranz JÁ De Santis M Bamias A Kikuchi E Del Muro XG. Atezolizumab plus chemotherapy versus placebo plus chemotherapy in untreated locally advanced or metastatic urothelial carcinoma (IMvigor130): final overall survival analysis results from a randomised, controlled, phase 3 study. Lancet Oncol. (2024) 25:29–45. doi: 10.1016/S1470-2045(23)00540-5 38101433

[38] Powles T Csőszi T Özgüroğlu M Matsubara N Géczi L Cheng SY. Pembrolizumab alone or combined with chemotherapy versus chemotherapy as first-line therapy for advanced urothelial carcinoma (KEYNOTE-361): a randomised, open-label, phase 3 trial. Lancet Oncol. (2021) 22:931–45. doi: 10.1016/S1470-2045(21)00152-2 34051178

[39] Flaig TW Spiess PE Abern M Agarwal N Bangs R Buyyounouski MK. NCCN guidelines^®^ Insights: bladder cancer, version 3.2024. J Natl Compr Canc Netw. (2024) 22:216–25. doi: 10.6004/jnccn.2024.0024 38754471

[40] Powles T van der Heijden MS Castellano D Galsky MD Loriot Y Petrylak DP. Durvalumab alone and durvalumab plus tremelimumab versus chemotherapy in previously untreated patients with unresectable, locally advanced or metastatic urothelial carcinoma (DANUBE): a randomised, open-label, multicentre, phase 3 trial. Lancet Oncol. (2020) 21:1574–88. doi: 10.1016/S1470-2045(20)30541-6 32971005

[41] Matsubara N de Wit R Balar AV Siefker-Radtke AO Zolnierek J Csoszi T. Pembrolizumab with or without lenvatinib as first-line therapy for patients with advanced urothelial carcinoma (LEAP-011): A phase 3, randomized, double-blind trial. Eur Urol. (2024) 85:229–38. doi: 10.1016/j.eururo.2023.08.012 37778952

[42] Fu Z Li S Han S Shi C Zhang Y. Antibody drug conjugate: the “biological missile” for targeted cancer therapy. Signal Transduct Target Ther. (2022) 7:93. doi: 10.1038/s41392-022-00947-7 35318309 PMC8941077

[43] Dumontet C Reichert JM Senter PD Lambert JM Beck A. Antibody-drug conjugates come of age in oncology. Nat Rev Drug Discov. (2023) 22:641–61. doi: 10.1038/s41573-023-00709-2 37308581

[44] Khoury R Saleh K Khalife N Saleh M Chahine C Ibrahim R. Mechanisms of resistance to antibody-drug conjugates. Int J Mol Sci. (2023) 24:9674. doi: 10.3390/ijms24119674 37298631 PMC10253543

[45] Wei Q Li P Yang T Zhu J Sun L Zhang Z. The promise and challenges of combination therapies with antibody-drug conjugates in solid tumors. J Hematol Oncol. (2024) 17:1. doi: 10.1186/s13045-023-01509-2 38178200 PMC10768262

[46] You X Zhu C Yu P Wang X Wang Y Wang J. Emerging strategy for the treatment of urothelial carcinoma: Advances in antibody-drug conjugates combination therapy. BioMed Pharmacother. (2024) 171:116152. doi: 10.1016/j.biopha.2024.116152 38228034

[47] Hoimes CJ Flaig TW Milowsky MI Friedlander TW Bilen MA Gupta S. Enfortumab vedotin plus pembrolizumab in previously untreated advanced urothelial cancer. J Clin Oncol. (2023) 41:22–31. doi: 10.1200/JCO.22.01643 36041086 PMC10476837

[48] Maguire WF Lee D Weinstock C Gao X Bulik CC Agrawal S. FDA approval summary: enfortumab vedotin plus pembrolizumab for cisplatin-ineligible locally advanced or metastatic urothelial carcinoma. Clin Cancer Res. (2024) 30:2011–6. doi: 10.1158/1078-0432.CCR-23-3738 PMC11096048 38441576

[49] Brave MH Maguire WF Weinstock C Zhang H Gao X Li F. FDA approval summary: enfortumab vedotin plus pembrolizumab for locally advanced or metastatic urothelial carcinoma. Clin Cancer Res. (2024) 30:4815–21. doi: 10.1158/1078-0432.CCR-24-1393 PMC11530298 39230571

[50] Powles T Bellmunt J Comperat E De Santis M Huddart R Loriot Y. ESMO Clinical Practice Guideline interim update on first-line therapy in advanced urothelial carcinoma. Ann Oncol. (2024) 35:485–90. doi: 10.1016/j.annonc.2024.03.001 38490358

[51] Sheng X Yan X Wang L Shi Y Yao X Luo H. Disitamab vedotin, a novel humanized anti-HER2 antibody-drug conjugate (ADC), combined with toripalimab in patients with locally advanced or metastatic urothelial carcinoma: An open-label phase 1b/2 study. J Clin Oncol. (2023) 41:4566. doi: 10.1200/JCO.2023.41.16_suppl.4566

[52] Loriot Y Petrylak DP Rezazadeh Kalebasty A Fléchon A Jain RK Gupta S. TROPHY-U-01, a phase II open-label study of sacituzumab govitecan in patients with metastatic urothelial carcinoma progressing after platinum-based chemotherapy and checkpoint inhibitors: updated safety and efficacy outcomes. Ann Oncol. (2024) 35:392–401. doi: 10.1016/j.annonc.2024.01.002 38244927

[53] Loriot Y Matsubara N Park SH Huddart RA Burgess EF Houede N. Erdafitinib or chemotherapy in advanced or metastatic urothelial carcinoma. N Engl J Med. (2023) 389:1961–71. doi: 10.1056/NEJMoa2308849 37870920

